# Diagnosing Basal Cell Carcinoma of the Vulva: A Case Report and Review of the Literature

**DOI:** 10.7759/cureus.20791

**Published:** 2021-12-29

**Authors:** Justin C Rudd, Changzhao Li, Rasam Hajiannasab, Jimmy Khandalavala, Poonam Sharma

**Affiliations:** 1 Department of Biomedical Sciences, Creighton University, Omaha, USA; 2 Department of Pathology, Creighton University, Omaha, USA; 3 Department of Obstetrics and Gynecology, Creighton University, Omaha, USA

**Keywords:** vulvar cancer, basal cell carcinoma immunohistochemistry, basal cell carcinoma diagnosis, basal cell carcinoma histopathology, vulvar basal cell carcinoma

## Abstract

Basal cell carcinoma (BCC) is a highly prevalent epidermal neoplasm that most commonly occurs in regions of sun-exposed skin, though rare cases arise in sun-protected areas. BCCs of the vulva account for a small fraction of cases and can be mistaken for other cutaneous genital pathologies on clinical examination. Here we report a case of vulvar BCC that presented as a firm, tender bilateral lesion of the mons pubis and was diagnosed by histopathology and immunostaining for classical BCC markers.

## Introduction

Basal cell carcinoma (BCC), a malignancy of epidermal keratinocytes, is the most prevalent human cancer, with more than three million cases reported in the U.S. every year [1]. It is estimated that only 1-2% of these BCCs involve the vulvar epidermis [2-5]. While BCCs have an excellent prognosis and metastasize infrequently [4], local tissue damage caused by vulvar BCCs can impart significant physical discomfort, cosmetic distress, and impair sexual function. Variable gross presentation of these vulvar lesions frequently leads to improper diagnosis and delayed biopsy [3]. Upon biopsy, these lesions may be easily diagnosed both through histopathology using H&E staining and immunohistochemical detection of BCC markers. Wide local excision of vulvar BCCs is typically curative, though Mohs micrographic surgery (MMS) may be more successful in preventing local recurrence. In this report, we highlight the common clinical and histopathological presentation of this rare BCC type in order to facilitate initial diagnosis and expedient removal.

## Case presentation

A 51-year-old (G3P2012) female patient presented to the clinic with vaginal itching, irritation, and a tearing sensation near the top of the vaginal area that had been present for one year. She reported that her physical discomfort had waxed and waned over that time but had worsened over the last several months. She denied excessive vaginal odor, general pelvic pain, fever, or chills and had no concerns for sexually transmitted infection. She stated that her discomfort was worsened by physical activity and moisture upon daily swimming. Physical examination revealed a 1.5 cm firm bilateral vulvar lesion with shallow red ulcerations at the lower mons pubis.

**Figure 1:**
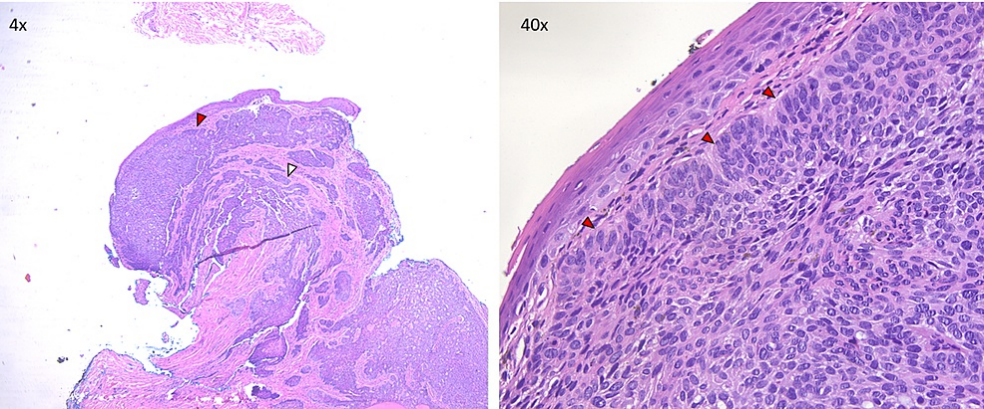
H&E staining of biopsy specimen. In the 4× image (left), keratinocyte nests can be observed in the papillary (red arrowhead) and reticular (white arrowhead) dermis. In the 40× image (right), peripheral palisading cells (red arrowheads) are evident in a dermal keratinocyte nest. H&E: hematoxylin and eosin.

An initial 3 mm Keyes punch (Miltex Instruments-Integra LifeSciences, Princeton, NJ, USA) biopsy of the lesion was performed and sent for both virologic and pathologic testing. Herpes simplex virus 1 and 2 (HSV1/2) testing by polymerase chain reaction (PCR) was negative. Microscopically, hematoxylin and eosin (H&E) staining showed both papillary and reticular dermal nests composed of hyperchromatic keratinocytes with peripheral cell palisading (Figure 1).

**Figure 2:**
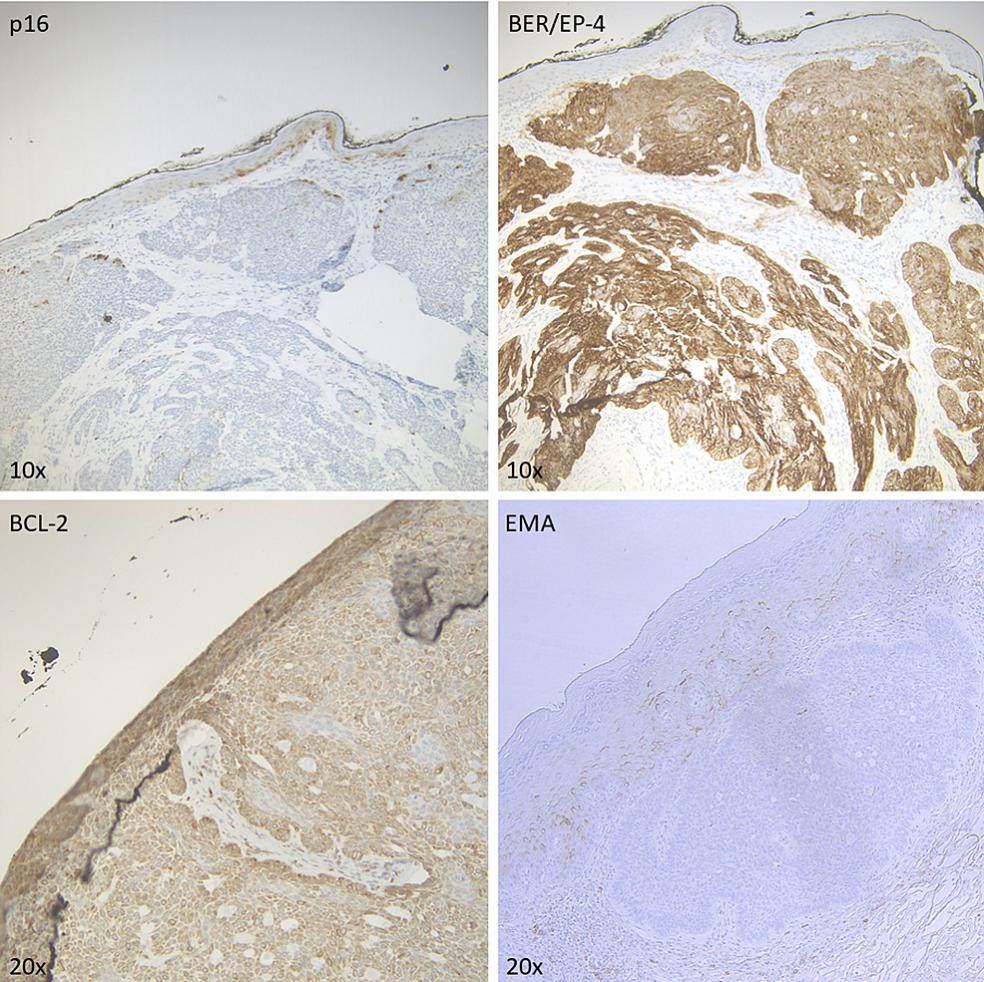
Immunohistochemical staining of biopsy specimen. Immunohistochemical staining of the biopsy specimen revealed patchy positive p16 signal, diffuse BER/EP-4 and BCL-2 positivity, and no EMA signal. BCL-2: B-cell lymphoma 2, EMA: epithelial membrane antigen.

Keratinocyte nests showed uniform membranous immunohistochemistry (IHC) for epithelial cell adhesion molecule (EPCAM/BER-EP4) and diffuse cytoplasmic staining for B-cell lymphoma 2 (BCL2) (Figure 2). These histopathologic findings confirmed the diagnosis of superficial and nodular type vulvar BCC.

Because the tumor was present at the peripheral margins of the initial biopsy, the patient returned for a wider excision of the lesion. Intraoperative findings revealed two distinct lesions; one 1 cm × 0.5 cm lesion on the right mons superior to the clitoral hood and one 3 mm × 3 mm lesion on the left side of the mons. An excision biopsy of the lesion involving the right mons was sent for histopathology, which confirmed superficial and nodular BCC with an estimated invasive depth of 1.6-2 mm. Additionally, the tumor was present at the superior margin and was 0.3 mm from the inferior margin, and 1.8 mm from the deep margin. No perineural or lymphovascular invasion was observed. Additional IHC was performed on the excisional biopsy specimen and showed patchy positive p16 signal in keratinocyte nests and no epithelial membrane antigen (EMA) signal in these cells (Figure 2). This immunoprofile supported the diagnosis of vulvar BCC. A punch biopsy of the small lesion on the left mons showed a benign capillary hemangioma.

## Discussion

While BCC is the most prevalent human cancer, most lesions develop on sun-exposed skin, and less than 1% of cases arise from the vulvar epidermis. Although the etiology of BCC in sun-protected areas like the vulva is poorly understood, animal models suggest that patched 1 (PTCH1) mutant hair follicle stem cells can give rise to BCC in the absence of ultraviolet (UV) irradiation [6,7]. This suggests that mutations in Sonic hedgehog (Shh) pathway components derived from non-UV insults, like chronic inflammation or exposure to arsenic or ionizing radiation, may be able to drive BCC development in sun-protected areas. Alternatively, hereditary Shh pathway mutations, like those observed in Gorlin’s syndrome, may underly non-UV-related BCCs. Evidence of pre-existing inflammatory conditions, environmental mutagen exposure, and underlying hereditary genodermatoses in vulvar BCCs is currently sparse [3-5], although this may be due to infrequent reporting. Deep targeted sequencing of known BCC driver genes in BCC tissue derived from sun-protected sites could help identify signatures of the exogenous or endogenous mutational processes that produce these rare tumors.

In addition to their poorly defined etiology, vulvar BCCs are challenging to diagnose clinically, owing both to non-specific clinical symptoms such as pain, pruritus and inflammation as seen in our patient, and the variable physical appearance of the lesion itself [4]. It is not uncommon for vulvar BCC to be initially misdiagnosed as either a general inflammatory skin condition such as psoriasis or dermatitis [3], or as an unrelated neoplastic disease like human papillomavirus (HPV)-associated squamous cell carcinoma (SCC), lichen sclerosis, or extramammary Paget disease [8].

Efforts to improve initial diagnosis have included database mining to generate a typical clinical presentation and the utilization of advanced imaging techniques during an assessment. A recent query of the surveillance, epidemiology, and end results (SEER) database found that among instances of vulvar BCC where the primary site was specified (n=347), the labia majora accounted for nearly 90% of the cases [5]. This preference for localization on the labia majora was also validated in a recent case series [9]. These cases predominantly affect elderly (median age 73-74) Caucasian women, and the mean tumor size is <2.0 cm [5,9]. This large size at diagnosis is in line with previous case reports [8]. These findings suggest that our patient likely represents a typical clinical presentation of this rare BCC type. The use of dermoscopy and reflectance confocal microscopy (RCM) for examination of vulvar lesions may also improve initial diagnostic accuracy. On dermoscopy, non-pigmented vulvar BCCs can display characteristic arborizing telangiectasia, ovoid nests, and whitish markings [10,11] that are difficult to visualize without a dermoscope. Additionally, RCM allows for visualization of nodular keratinocyte nests in the dermis that cannot be seen from the skin surface [12]. Reports of dermoscopy and RCM for vulvar BCC diagnosis are rare in the literature. However, the techniques clearly provide a diagnostic advantage for practiced users.

Upon biopsy, vulvar BCC is histopathologically similar to BCC on sun-exposed skin. Superficial and nodular subtypes, which account for the majority of vulvar BCC cases [5] and were observed in the patient case reported here, are characterized by the presence of dermal keratinocyte nests that display peripherally palisading basal cells. Palisading cells often retract from adjacent stroma to create “retraction spaces” [13]. Atypical disease subtypes including basosquamous, infiltrative, and infundibulocystic vulvar BCCs have been reported sparingly in the literature [3,4,14], although recent data suggest that tumor subtype is less prognostic of disease recurrence than tumor size at biopsy [5,9]. This highlights the importance of early diagnosis. Additionally, while we report a vulvar lesion that presented with a typical BCC IHC profile (BCL2 positive, BER-EP4 positive, patchy p16 positive), immunostaining is infrequently implemented for the diagnosis of vulvar BCC. BER-EP4 and p16 IHC may be particularly useful for vulvar BCC diagnosis as these tumors are often mimicked by HPV-related basaloid SCC, which are more prevalent and carry a worse prognosis. These SCCs stain BER-EP4 negative and diffusely p16 positive in a “block pattern,” while the BCC typically stains BER-EP4 positive and displays patchy p16 signal [15,16].

The therapeutic strategy for vulvar basal cell carcinoma most commonly includes excisional biopsy, as was the case for the patient described above, or wide local excision [3,4]. The use of Mohs micrographic surgery (MMS) has been reported to improve the rate of recurrence of vulvar BCC, though the sample size is still very small, with only 15 cases being reported [4,14,17]. Overall, these surgical therapies are largely curative, with an estimated 5% of cases recurring locally, typically due to incomplete excision [4]. In a comprehensive review of vulvar BCC cases indexed in Medline and Embase, Renati and colleagues found that only 2.7% of cases (n=446) metastasized. Among metastatic lesions, spread via inguinal lymph nodes was the most common [4]. Currently, there are no guidelines for the staging of BCC of the vulva. According to the cancer protocol of the College of American Pathologists (CAPs), SCCs confined to the vulva with a depth of stromal invasion of 1.0 mm or less are staged as pT1a, whereas those with an invasion of more than 1.0 mm are staged as pT1b. More data are needed to see whether the depth of invasion is critical for reporting vulvar BCCs in terms of prognosis. However, we routinely mention both the depth of invasion and the margin status in the diagnostic report for better clinical management. Our patient presented here, thus, represents a typical treatment course, utilizing excision biopsy to remove a locally invasive vulvar BCC.

## Conclusions

Our patient presented to the clinic complaining of vaginal pruritus and pain stemming from a 1.5 cm bilateral, firm, ulcerated lesion of the lower mons pubis. Prompt Keyes punch biopsy and immunohistopathology were performed to make a diagnosis of vulvar BCC. We describe the use of an IHC panel for confirmation of BCC diagnosis (BER-EP4, p16, BCL2) that has been reported in the literature to improve correct differentiation between vulvar BCC and HPV-associated SCC of the vulva. We also draw attention to the benefit of dermoscopic and RCM imaging of vulvar BCC and ask that readers consider incorporating these techniques in workups of cutaneous vulvar lesions as they may prevent delays in biopsy and excision.
